# Crystal structures from the *Plasmodium *peroxiredoxins: new insights into oligomerization and product binding

**DOI:** 10.1186/1472-6807-12-2

**Published:** 2012-03-19

**Authors:** Wei Qiu, Aiping Dong, Juan C Pizarro, Alexei Botchkarsev, Jinrong Min, Amy K Wernimont, Tanya Hills, Raymond Hui, Jennifer D Artz

**Affiliations:** 1 Structural Genomics Consortium, University of Toronto, 101 College St., MaRS South Tower, Toronto, ON M5G 1L7, Canada

## Abstract

**Background:**

*Plasmodium falciparum *is the protozoan parasite primarily responsible for more than one million malarial deaths, annually, and is developing resistance to current therapies. Throughout its lifespan, the parasite is subjected to oxidative attack, so *Plasmodium *antioxidant defences are essential for its survival and are targets for disease control.

**Results:**

To further understand the molecular aspects of the *Plasmodium *redox system, we solved 4 structures of *Plasmodium *peroxiredoxins (Prx). Our study has confirmed *Pv*Trx-Px1 to be a hydrogen peroxide (H_2_O_2_)-sensitive peroxiredoxin. We have identified and characterized the novel toroid octameric oligomer of *Py*Trx-Px1, which may be attributed to the interplay of several factors including: (1) the orientation of the conserved surface/buried arginine of the NNLA(I/L)GRS-loop; and (2) the *C*-terminal tail positioning (also associated with the aforementioned conserved loop) which facilitates the intermolecular hydrogen bond between dimers (in an A-C fashion). In addition, a notable feature of the disulfide bonds in some of the Prx crystal structures is discussed. Finally, insight into the latter stages of the peroxiredoxin reaction coordinate is gained. Our structure of *Py*Prx6 is not only in the sulfinic acid (RSO_2_H) form, but it is also with glycerol bound in a way (not previously observed) indicative of product binding.

**Conclusions:**

The structural characterization of *Plasmodium *peroxiredoxins provided herein provides insight into their oligomerization and product binding which may facilitate the targeting of these antioxidant defences. Although the structural basis for the octameric oligomerization is further understood, the results yield more questions about the biological implications of the peroxiredoxin oligomerization, as multiple toroid configurations are now known. The crystal structure depicting the product bound active site gives insight into the overoxidation of the active site and allows further characterization of the leaving group chemistry.

## Background

There are at least 500 million clinical episodes of malaria annually with more than a million Africans dying each year, most of whom are children under 5 years of age [1]. The causative agent for the most lethal form of malaria is a protozoan parasite, *Plasmodium falciparum*, while *P. vivax *causes a less severe form, *P. knowlesi *is responsible for macaque malaria (but it can also infect humans [2,3]), and *P. yoelii *and *berghei *infect rodents. *Plasmodium *parasites are frequently subject to oxidative attack, for example, in the erythrocyte from H_2_O_2 _release during heme metabolism and from NO and reactive oxygen species (ROS) generation during the host immune response [4,5]. In addition, oxidative stress is sustained during the sexual maturation of the parasite within the *Anopheles *mosquito midgut and salivary gland prior to transmission [6,7]. As such, *Plasmodium *antioxidant defences are essential to its survival, and thus are expected to be targets for the effective control of the disease [8,9].

Interestingly, neither the *Plasmodium *parasites nor the trypanosomes contain a catalase or a selenocysteine-containing glutathione peroxidase (GPx), which are enzymes notably efficient for the detoxification of hydroperoxides [10,11]. *Plasmodium *does however possess 2 superoxide dismutases, 6 proteins homologous to thiol-dependent peroxidases, and a glutathione-*S*-transferase (GST). The GST has only weak GSH peroxidase activity, but it might contribute significantly to the antioxidant capacity of the parasite due to its high concentration [12]. Of those homologous to the thiol-dependent peroxidases, there is the GPx-like thioredoxin peroxidase, which is a non-selenocysteine GPx known to be significantly less active than its selenium homologue [13]. The 5 remaining thiol-dependent peroxidase homologues identified in *Plasmodium *include thioredoxin peroxiredoxin 1 and 2 (Trx-Px1 and Trx-Px2) from the peroxiredoxin subfamily Prx1, a 1-Cys peroxiredoxin (1-Cys Prx) from the Prx6 subfamily, antioxidant protein (AOP) from subfamily Prx5, and a very recently characterized nuclear peroxiredoxin (*Pf*nPRx) [14] (Table 1). Interestingly, peroxiredoxins have recently been implicated in a different role, namely as a non-transcriptional rhythmic marker, indicative of the circadian clock [19]. Various strategies have been used to classify the members of the peroxiredoxin family including a phylogenetic tree analysis that categorizes them into 6 subfamilies (Prx1, Prx6, Prx5, Trx-Px, BCP, and AhpE), each of which may include the mechanistically distinct 1-Cys and 2-Cys peroxiredoxins [20,21].

**Table 1:** *Plasomodium *peroxiredoxin orthologues and corresponding PDB codes for solved structures

Subfamily	Name	Mechanistic Classification	Cellular Location	PlasmoDB ID	PDB ID	Reference
Prx1	*Pf*Trx-Px1	2-Cys (C50, C170)	cytosolic	PF14_0368		[15]
	*Pv*Trx-Px1	2-Cys (C50, C170)	cytosolic	PVX_118545	(2H66, 2I81)	this work
	*Py*Trx-Px1	2-Cys (C50, C170)	cytosolic	PY00414	(2H01)	this work
	*Pb*Trx-Px1	2-Cys (C50, C169)	cytosolic	PB000037.01.0		
	*Pk*Trx-Px1	2-Cys (C50, C170)	cytosolic	PKH_126740		
	*Pf*Trx-Px2	2-Cys (C67, C187)	mitochondrial	PFL0725w	(2C0D)	[15,16]
	*Pv*Trx-Px2	2-Cys (C67, C187)	mitochondrial	PVX_123435		
	*Py*Trx-Px2	2-Cys (C67, C187)	mitochondrial	PY02747		
	*Pb*Trx-Px2	2-Cys (C59, C179)	mitochondrial	PB001545.02.0		
	*Pk*Trx-Px2	2-Cys (C67, C187)	mitochondrial	PKH_143220		
Prx6	*Pf*Trx-Px1	1-Cys (C47)	cytosolic	PF08_0131		
	*Pv*Trx-Px1	1-Cys (C47)	cytosolic	PVX_093630		
	*Py*Trx-Px1	1-Cys (C47)	cytosolic	PY04285	(3TB2)	this work
	*Pb*Trx-Px1	1-Cys (C47)	cytosolic	PB000600.02.0		
	*Pk*Trx-Px1	1-Cys (C47)	cytosolic	PKH_011610		
Prx5	*Pf*Trx-Px1	1-Cys (C117)	apicoplast	MAL7P1.159	(1XIY)	[17]
	*Pv*Trx-Px1	1-Cys (C114)	apicoplast	PVX_081760		
	*Py*Trx-Px1	1-Cys (C122)	apicoplast	PY01475		
	*Pb*Trx-Px1	1-Cys (C28)	apicoplast	PB000177.01.0		
	*Pk*Trx-Px1	1-Cys (C114)	apicoplast	PKH_021360		
BCP	*Pf*nPrx	1-Cys (C56)	nuclear	PF10_0268		[14,18]
	*Pv*nPrx	1-Cys (C52)	nuclear	PVX_111355		
	*Py*nPrx	1-Cys (C52)	nuclear	PY03834		
	*Pb*nPrx	1-Cys (C61)	nuclear	PBANKA_051140		
	*Pk*nPrx	1-Cys (C52)	nuclear	PKH_061160		

Abbreviations include: *Pf, P. falciparum*; *Pv, P. vivax*; *Py, P. yoelii*; *Pb, P. berghei*; and *Pk, P. knowlesi*. Cellular location from experimental result, from result of orthologue, or from predictive targeting sequences. Mechanistic classification from experiment or based on experimental result and sequence alignment of orthologue

All peroxiredoxins contain a conserved cysteine residue at the *N*-terminus that is referred to as the peroxidatic cysteine (C_P_). During catalysis, it is oxidized by the ROS substrate (generally H_2_O_2 _or an alkyl hydroperoxide) to sulfenic acid (Cys-S-OH). Typical 2-Cys Prx contain 2 conserved cysteines, including the C_P _and a *C*-terminal cysteine (termed the resolving Cys (C_R_)). During catalysis, the C_P _sulfenic acid reacts with the C_R _of the adjacent monomer to form the intermolecular disulfide of the homodimer that is subsequently reduced by another (undetermined) thiol. In 1-Cys Prx, the C_P _sulfenic acid is directly reduced by an unidentified redox partner.

Building on the dimer formation, the 2-Cys Prx enzymes organize themselves into higher order oligomers, such as decamers, which have higher peroxidase activity. Formation of the higher order oligiomers is dependent on the redox state of C_P _(and C_R_), as well as other factors [22,23]. Both Trx-Px1 and Trx-Px2 have been identified as typical 2-Cys Prx enzymes; and a crystal structure of *P. falciparum *Trx-Px2 (PDB ID: 2C0D) has been published [16]. Distinguishing these two *Plasmodium *thioredoxin peroxidases is their cellular location, as Trx-Px1 is predicted to be cytosolic and Trx-Px2 has a mitochondrial targeting sequence [15]. Both features were recently confirmed [24]. Like the *Plasmodium *1-Cys Prx from subfamily Prx6, AOP is also a 1-Cys Prx; and the *P. falciparum *AOP structure has been solved (PDB ID: 1XIY) [17]. AOP is thought to be an apicoplast enzyme due to its *N*-terminal signal motif, while the other *Plasmodium *1-Cys Prx is cytosolic. Both predictions were recently confirmed experimentally [24]. Prx enzymes are highly expressed in *Plasmodium *(0.5% of cellular protein) and have been predicted from competitive kinetic analysis with human cells to be responsible for the reduction of 90% of mitochondrial H_2_O_2 _and nearly 100% of cytoplasmic H_2_O_2 _[25].

In order to further the understanding of the molecular details of the *Plasmodium *redox system, we solved the crystal structures of Trx-Px1 from *P. vivax *(*Pv*Trx-Px1) in the reduced and oxidized forms, Trx-Px1 from *P. yoelii *(*Py*Trx-Px1) in the oxidized form, and a 1-Cys Prx from *P. yoelii *(termed *Py*Prx6, herein) with C_P _oxidized to the sulfinic acid and with glycerol bound within the active site pocket. In addition, we have structurally confirmed and characterized the *Pv*Trx-Px1 as a H_2_O_2_-sensitive peroxiredoxin; *Py*Trx-Px1 as forming an octamer (instead of the typical decamer and dodecamer arrangements); and *Py*Prx6 as a product bound complex revealing some interesting features of these enzymes.

## Results

### Expression and initial characterization of Trx-Px1 from *P. falciparum, P. vivax, P. yoelii*, and *P. knowlesi *and Prx6 from *P. yoelii*

Constructs of the Trx-Px1 enzymes from *P. falciparum, P. vivax, P. yoelii*, and *P. knowlesi *were expressed and purified as described previously [26]. *Py*Prx6 was also expressed in Studier auto-induction media [27]. All were full length constructs except *Py*Trx-Px1 which was also expressed for crystallization with a 6-residue truncation at the *N*-terminus. According to our mass spectroscopic analysis, all of our purified Trx-Px1 enzymes were disulfide-linked dimers (Table 2). As verified by mass spectroscopy, each of the 4 purified Trx-Px1 enzymes could be completely reduced using 20 mM dithiothreitol (Table 2).

**Table 2:** Mass spectroscopy of reduced and oxidized Plasmodium Trx-Px-1 orthologues

	Expected MW of Reduced Monomer	Expected MW of Oxidized Dimer	Purified Enzyme (Da)	Purifed Enzyme + 20 mM DTT (Da)
*Pf*Trx-Px1	23702.08	47400.12	47456.56 (99%+) 23729.32 (trace) ^a^	23730.96

*Pv*Trx-Px1	23567.94	47131.84	47133.20 (major) 23568.94 (minor)	23568.77

*Py*Trx-Px1	23534.86	47065.68	47066.49 (99%+) 23533.70 (trace)	23533.70

*Py*Trx-Px1: Q7-L195	23234.40	46468.76	46470.60(99%+) 23234.49 (trace)	23235.18

*Pk*Trx-Px1	24225.58	48447.12	48449.43 (99%+) 24226.10 (trace)	24226.38

The calculated MW in Da is determined from the amino acid sequence and includes the His_6_-tag incorporated into our constructs. The expected monomer MW is determined from the calculated monomer MW and subtracts the known *E. coli *post translational modification, namely clipping of the *N*-terminal Met (-131.19) of the His_6_-tag [26]. The expected MW of the oxidized dimer is calculated by doubling the expected MW weight of the monomer (clipping of the N-terminal Met accounted for) and subtracting 4 H (-4.04). ^a^The MW of the purified monomeric *Pf*Trx-Px-1 is off by 28 Da (and the dimer is off by twice this) which may be accounted for by one of the following: addition of ethyl addition, *N, N*-dimethylation of Arg or Lys, 2,4 bis-Trp-6,7-dione formation, or addition of formaldehyde (CHO)

By analytical gel filtration, all of the purified Trx-Px1 constructs (oxidized form and at high μM concentrations) eluted primarily as the higher order oligomer (*i.e*. octamer or decamer or dodecamer) (data not shown). Oligomerization has been shown to be dependent on numerous factors including ionic strength, pH, concentration of divalent metals, and most importantly redox state [28]. In our gel filtration, a small amount of presumably aggregated protein was followed by the oligomeric protein around (corresponding to a calculated mass of 272 or 314 kDa). In the case of *Pf*Trx-Px1, some presumably dimeric protein was also observed, which was not noticed in previous gel filtration analyses of *Pf*Trx-Px1 by Akerman and coworkers. [29]. These authors only observed higher order oligomers corresponding to 400 and 250 kDa for *Pf*Trx-Px-1 and further detected the (α_2_)_5 _quaternary form by electron microscopy.

*Py*Prx6 was expressed in two ways: *Py*Prx6 expressed from our typical protocol was purified in the reduced form according to mass spectroscopy (27189.1 Da) without addition of exogenous reducing agents (with the expected molecular weight after cleavage of the *N*-terminal Met weight being 27188.2 Da), while *Py*Prx6 expressed from Studier auto-induction media was purified in the sulfinic acid form (see below). From a calibrated gel filtration column, the enzyme eluted at 220 mL (corresponding to a calculated gel filtration mass of 62 kDa) which is consistent with the expected behaviour of a dimer.

### Crystal structures of Trx-Px1 from *P. vivax *and *P. yoelii*

**Figure 1:**
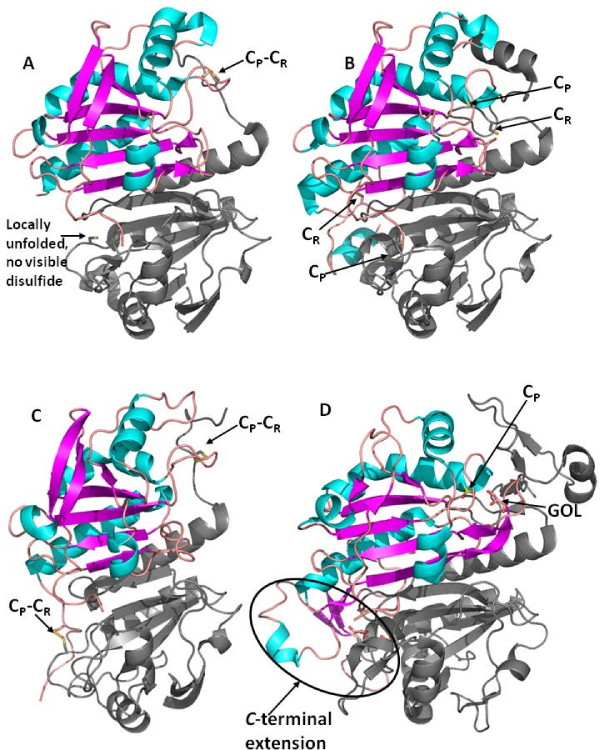
**Structures of the dimeric units of the *Plasmodium *peroxiredoxins**. The structures of the dimeric units of the peroxiredoxins with the C_P _and C_R _thiol side chains are shown to display the secondary structure in one of the monomers with α-helices shown in blue, β-sheets shown in pink, and sulfur and oxygen atoms displayed in yellow and red, respectively. **(A) ***Pv*Trx-Px1_ox is shown with only one disulfide visible, as the second one is not visible due to the lack of structure at the *C*-terminus (thus C_R_) of the monomer shown with secondary structure colours. **(B) ***Pv*Trx-Px1_red with all 4 reduced thiols clearly visible. **(C) ***Py*Trx-Px1_ox with the 2 disulfides clearly displayed; and **(D) ***Py*Prx6 is shown with its sulfinic acid active site cysteine and with glycerol bound.

The crystal structures of *P. vivax *Trx-Px1 were solved in both the reduced (*Pv*Trx-Px1_red, PDB ID: 2I81) and oxidized forms (*Pv*Trx-Px1_ox, PDB ID: 2H66) at 2.45 Å and 2.5 Å, respectively (Figure 1). Aside from these 2 structures, to date only rat and *Salmonella typhimurium *Prx1 subfamily structures have been solved in both fully reduced and oxidized (as the disulfide) redox states [21]. In comparison to other solved structures, *Pv*Trx-Px1 is most similar (52% sequence identity) to *Pf*Trx-Px2 [16]. It has 85% sequence identity with *Pf*Trx-Px1 and 47% identity to its closest human orthologues. Herein, the oxidized form of *Py*Trx-Px1 has also been solved to 2.3 Å (PDB ID: 2H01) (Figure 1C).

The structures of each (*Pv*Trx-Px1_red, *Pv*Trx-Px1_ox, and *Py*Trx-Px1_ox) contain the typical thioredoxin-fold found in known peroxiredoxins. There is a central 7-stranded β-sheet comprised of β2-β1-β5-β4-β3-β6-β7 with β1 and β6 running anti-parallel relative to the other strands. This β-sheet is sandwiched by α1 and α4 on one side and α2, α3, and α5 on the other side. The root mean square deviation (rmsd) between *Pv*Trx-Px1_ox, and *Py*Trx-Px1_ox is 0.91 Å when superimposing over the monomer encompassing Cα's from residues 2 to 177. The remaining residues of the *C*-terminus are disordered (from 178 to 195) in the *Pv*Trx-Px1_ox structure.

**Figure 2:**
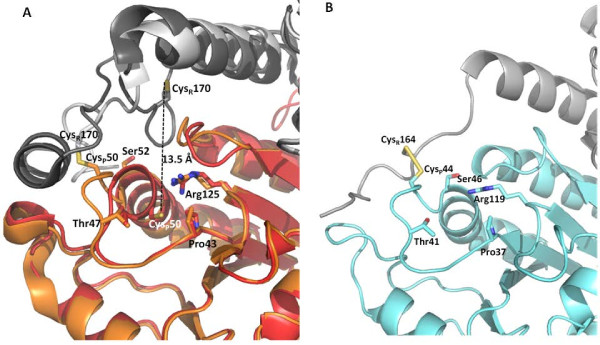
***Pv*Trx-Px1 and *Py*Trx-Px1 active sites**. **(A) **Active sites of *Pv*Trx-Px1_red and *Pv*Trx-Px1_ox are shown in red/dark grey and orange/light grey, respectively. Note the positioning of Pro43, Thr47, and Arg125 are unchanged between the reduced and oxidized forms. The dramatic change of the active site C_P _in the reduced form (red) is shown as untwisting of the helix to meet the C_R _from its dimeric partner. As well, the formation of the disulfide results in the disruption of the final *C*-terminal α-helix. Note that in the reduced form the C_P _and C_R _are separated by 13.5 Å. **(B) **Active site of *Py*Trx-Px1_ox is shown for comparison.

In *Pv*Trx-Px1_red structure, the C_P _residue (Cys50 for *P. vivax*) is located at the first turn of helix α2 at the end of a narrow accessible channel formed by a loop-helix motif and surrounded by 3 conserved residues Pro43, Thr47, and Arg125 (Figure 2A, red/dark grey). The pyrrolidine ring of Pro43 limits solvent accessibility and protects the reactive cysteinyl sulfenic acid from further oxidation during catalysis. The distance between Cys50 and the corresponding C_R _thiol from its dimeric partner is 13.5 Å in the reduced form. In both the oxidized structures of *Pv*Trx-Px1_ox and *Py*Trx-Px1_ox, the α2 helix is locally unfolded (LU) around the C_P_, such that the α2 helix begins after the C_P _with Ser52 (coloured in orange in Figure 2A for *Pv *and *Py*: *Pv*Trx-Px1_ox) or Ser46 (Figure 2B for *Py*Trx-Px1_ox). Some conserved residues that form the active site in the reduced structure are in the same positions and orientations in both the oxidized structures (Pro43, Thr47, and Arg125 for PvTrx-Px1, Figure 2A, orange/light grey and Pro37, Thr41, and Arg119 for *Py*Trx-Px1, Figure 2B). The C_P _has rotated to the surface as part of a highly exposed loop; and S_P _(sulfur of C_P_) is engaged in a disulfide bond with S_R _(sulfur of C_R_) of the enzyme's dimeric partner forming a domain swapped homodimer.

### *Pv*Trx-Px1 and *Py*Trx-Px1 are H_2_O_2_-sensitive peroxiredoxins

**Figure 3:**
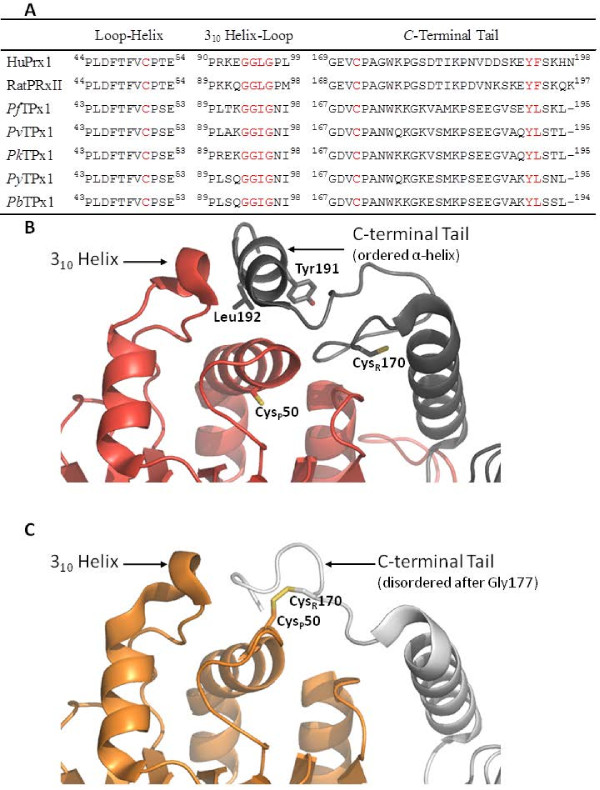
***Pv*Trx-Px1 is H_2_O_2_-sensitive**. **(A) **Comparison of the sequences of the H_2_O_2_-sensitive mammalian peroxiredoxins to the *Plasmodium *peroxiredoxins discussed herein. **(B) **&** (C) **Active sites of the reduced and oxidized forms of *Pv*Trx-Px1 exemplifying the features described in the text of the H_2_O_2_-sensitive peroxiredoxins.

The 3 sequence motifs that define the H_2_O_2_-sensitive peroxiredoxins include: (1) the conserved loop-helix from Pro43 to Glu53 surrounding the C_P_; (2) a 3_10 _helix-loop from Pro89 to Ile98; and (3) the 29 *C*-terminal residues from Gly167 including C_R _and conserved bulky residues [30]. The loop-helix is completely conserved among human, rat, and *Plasmodium *peroxiredoxins. However, the *Plasmodium *enzymes are a slight variation of 3_10 _helix-loop and *C*-terminal tail motifs with sequences including ^93^GGIG^96 ^and ^191^YL^192^, respectively, instead of the typical GGLG and YF (Figure 3A). Upon addition of 500 μM to 5 mM H_2_O_2 _to reduced (by DTT) *Pf*Trx-Px1, *Pv*Trx-Px1, *Pk*Trx-Px1, or *Py*Trx-Px1 enzymes, additions of 2 and 3 oxygen atoms were observed by mass spectroscopy, confirming that these enzymes are H_2_O_2_-sensitive. Structurally, the first loop-helix and the *C*-terminal arm of *P. falciparum *were predicted by modelling to undergo the same structural rearrangement during catalysis as the mammalian peroxiredoxins [31]. Figure 3B, 3C illustrate the structural changes that *Pv*Trx-Px1 does indeed undergo during catalysis further supporting its characterization as a H_2_O_2_-sensitive peroxiredoxin. Although predicted by Kawazu [31], to be fluid (*i.e*. structurally disordered) from Pro171 immediately following C_P_, the *C*-terminal tail is an ordered loop from Pro171 to Gly177 in the *Pv*Trx-Px1_ox structure. The *Py*Trx-Px1_ox structure also shows a similar arrangement of conserved residues, a 3_10 _helix-loop motif at ^90^PLSQGGIGNI^98^, and a *C*-terminal tail bearing a ^191^YL^192 ^motif (nearly identical to *Pv*Trx-Px1 that folds upon reduction into a loop followed by an α-helix), so it is also expected to be a H_2_O_2_-sensitive peroxiredoxin (Figure 2B and 3A). The conserved ^191^YL^192 ^motif that is located on the α-helix close to the surface stabilizes the full-folded conformation. This motif therefore slows the resolution reaction and allows overoxidation by reaction with a second equivalent of peroxide. In contrast robust peroxiredoxins do not have residues protecting the C_P _and are quickly oxidized to the disulfide [30].

### Disulfide bonds in the *Pv*Trx-Px1_ox and *Py*Trx-Px1_ox structures

**Figure 4:**
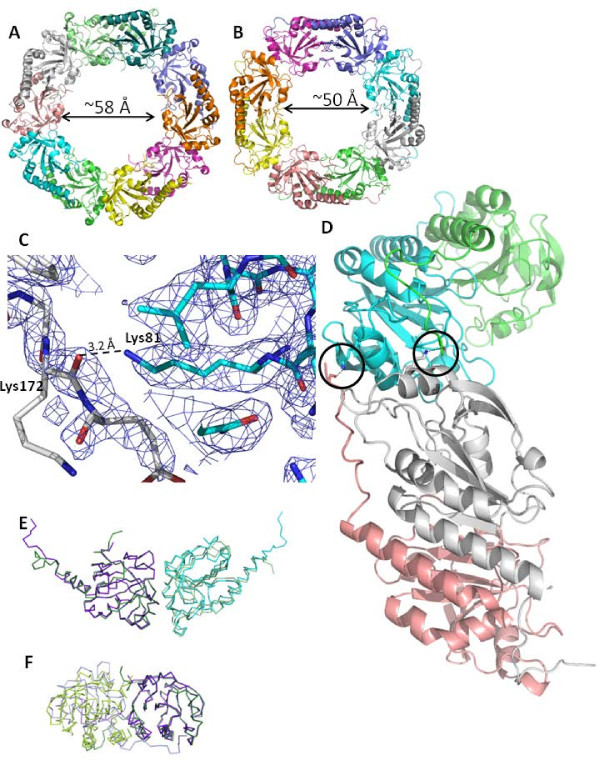
***Plasmodium *peroxiredoxin oligomeric structures**. Oligomeric structures of **(A) ***Pv*Trx-Px_ox and **(B) ***Py*Trx-Px1_ox showing the internal dimer. **(C) **The close-up view of a hydrogen bond at the A-type interface between the side chain of Lys81 from one monomer (cyan) and the main chain carbonyl of Lys172 from an adjacent molecule (grey) with the 2mFo-dFc electron map contoured at 1.0 σ (blue mesh). **(D) **Examples of the novel hydrogen bonding interactions of the *Py*Trx-Px_ox are circled in black. The panel is simplified to show only a tetramer for clarity, but indeed there are hydrogen bonding interactions across all of the A-type interfaces. Although the hydrogen bonding interaction is at the A-type interface, it is formed between distant chains in an A-C fashion (where A, B, C, and D chains are pink, grey, cyan, and green, respectively). For example, in the pink chain Lys172 carbonyl backbone is shown hydrogen bonding with the side chain of Lys81 from the cyan chain (A-C fashion) and in the grey chain Lys81 is hydrogen bonding with the main chain carbonyl of green Lys172 (B-D fashion). A comparison of the A-type interactions **(E) **and B-type interactions **(F) **between *Pv*Trx-Px1 and *Py*Trx-Px1 is shown. In **(E) **and **(F) **the monomer structures (dark green and purple) are structurally aligned (rmsd = 0.573 Å), so that upon comparison of the corresponding dimeric partners the difference in the interfaces are shown (*Pv*Trx-Px1 shown in light greens and *Py*Trx-Px1 shown in light blue/purple). A full alignment of the A-type dimers gives a rmsd = 1.065 Å, while a full alignment of the B-type dimers gives a rmsd = 3.137 Å.

Upon examination of each dimer of *Pv*Trx-Px1_ox decamer ((α_2_)_5 _oligomer) (Figures 1A and 4A), only 4 of a possible 10 disulfide bonds are clearly defined in the crystal structure. There is inadequate density to define the *C*-terminal tail from around the C_R _for the remaining residues, so that for several of the monomers only the side chain of C_P _but not that of C_R _is visible. In the case of the *Py*Trx-Px1_ox octamer ((α_2_)_4 _oligomer) (Figures 1C and 4B) (for which the data was collected at a home source) the Cys-S_P _to Cys-S_R _distances measure ~3 Å (notably, bond lengths at the resolution of these structures are derived from a combination of x-ray data and chemical constraints). There are no reports indicating that disulfide bonds are labile under the conditions used in our *Py*Trx-Px1 experiments. As expected, the *C*-terminal tails of *Pv*Trx-Px1 and *Py*Trx-Px1 have higher B-factors than the other parts of the molecule, indicative of a more fluid region and also of the apparent absence of detectable disulfide bonds in portions of the *Pv*Trx-Px1 structure and the distortion of the disulfide bond length in the *Py*Trx-Px1 structure. In previously published crystal structures of oxidized 2-Cys Prx, the Cys-S_P _to Cys-S_R _distance is also longer than expected for a disulfide bond (as the typical bond length is 2.05 for a disulfide). For example in the structure of a 2-Cys Prx from *Helicobacter pylori *(*Hp*AhpC) (PDB ID: 1ZOF), the Cys-S_P _to Cys-S_R _distances measurements range from 2.0 to 3.2 Å and in a *P. falciparum *2-Cys Prx (*Pf*Trx-Px2) (PDB ID: 2C0D) one of the disulfides is shown in two different orientations (2.0-2.2 Å) indicative of the structural flexibility of these structures, while the other measures at 2.6 Å. Our analysis using mass spectroscopy (already discussed) and the overall structural configurations (*i.e*. local unfolding about C_P _and at the *C*-terminus) both support *Pv*Trx-Px1_ox and *Py*Trx-Px1_ox being in the oxidized form making these long Cys-S_P _to Cys-S_R _distances not easily accounted for, yet prevalent in the Prx1 subfamily.

### Oligomeric organization of *Pv*Trx-Px1 and *Py*Trx-Px1

**Figure 5:**
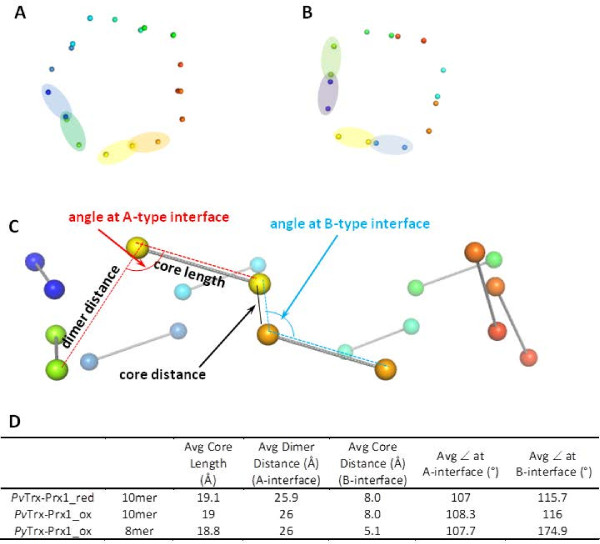
**Scheme describing the differences in oligomerization**. **(A) **The Cα's from 2 conserved leucine residues (*i.e*. Leu 102 and Leu139) are shown for *Pv*Trx-Px1. Two dimers are highlighted: the first comprised of blue and green monomers and second comprised of yellow and orange monomers. **(B) **The Cα's from 2 conserved leucine residues are shown (*i.e*. Leu96 and Leu133 in *Py*Trx-Px1). Two dimers are highlighted: the first comprised of green and purple monomers and second comprised of yellow and blue monomers. **(C) **The same spheres are shown from **(A) **from a different angle and scale with a grey line connecting the Lys's of the same monomer for clarity. The distances and angles calculated (by Pymol) in the next panel are defined. Note that these were made for the sole purpose of comparing the orientation of the monomers within the structure at the B-type interface and at the A-type interface. **(D) **Summary table showing the average distances and angles calculated for each of the measurements indicated in the previous panel. The standard deviations are not shown for clarity, but are less than 0.5% in each case.

Oligomeric peroxiredoxins are formed via at least 2 types of interactions: (1) B-type interactions where edge to edge associations of β7 strands of the central sheet meet to extend it into a 14-stranded β-sheet; and (2) A-type where the interface is a tip to tip association centred on helices α4 and α5 packing against helices α4 and α5 of the other chain [17]. The dimer interface of the *Pv*Trx-Px1_ox and *Py*Trx-Px1_ox (*i.e*. where the interactions resulting at the surfaces formed by the disulfide bond) is termed the B-type interaction face. Accordingly, the same dimeric unit is expected in the reduced structures. These dimers then associate via A-type interactions to form the higher order oligomers, which are typically decameric and dodecameric. *Pv*Trx-Px1_ox and *Pv*Trx-Px1_red arrange in a decameric fashion (according to our crystal structure), which from a survey of peroxiredoxin structures deposited to the PDB is the most common oligomerization. According to our results (namely a crystal structure and the gel filtration elution times characteristic of the higher order oligomer and not a dimer), the toroid *Py*Trx-Px1 structure is unique in that it is octameric (Figure 4B). *Pv*Trx-Px1_ox and *Pv*Trx-Px1_red decamers have internal diameters of ~58 Å, while *Py*Trx-Px1_ox octameric diameter is correspondingly smaller at ~50 Å. Li *et al. *reportedly solved the structure of another octameric peroxiredoxin (*Mycobacterium tuberculosis *AhpE), but this has been disputed as simply a crystallographic artifact, as predominately dimers were observed in gel filtration and the octameric interface is not extensive and does not involve the typical interfaces [32,33]. It could be argued that the octameric arrangement of *Py*Trx-Px1 is a result of the crystal packing of *Py*Trx-Px1_ox dimers; however, no dimers were observed in the gel filtration of the *Py*Trx-Px1_ox sample which was done at a high μM concentration comparable to crystallization experiments. Interestingly, there is a hydrogen bonding interaction in the *Py*Trx-Px1 structure between distant monomers and more specifically between adjacent dimers, where the *C*-terminal tail of one monomer crosses its dimeric partner to hydrogen bond to next monomer. Contributing to the stability of the octamer, the side chain of Lys81 is within hydrogen bonding distance (3.2 Å) of the backbone carbonyl of Lys172 (Figure 4C). Indeed, there are interactions between each of the pairs A-C, B-D, C-E, D-F, E-G, F-H, G-A, and H-B, as one would expect from symmetry (Figure 4D). Although these 2 residues are conserved in *Pv*Trx-Px1, there is no similar interaction in the *Pv*Trx-Px1_ox or *Pv*Trx-Px1_red structures (where the corresponding residues are Lys87 and Gly177). There is 83% sequence identity between the *P. yoelii *and *P. vivax *Trx-Px1 enzymes, so the differences in oligomeric state were not predictable. Previous reports have identified alterations of the B-type interface as conferring the different orders of oligomeric state, while the A-type interface remains constant [33]. An alignment of the Cα's of a monomer from the decameric *Pv*Trx-Px1_ox with a monomer from octameric *Py*Trx-Px1_ox (rmsd = 0.573 Å) is shown in Figure 4E, F to illustrate the overlap of each accompanying monomer in the A-type interface and B-type interface, respectively. In Figure 4E, the corresponding A-type interface dimer is shown for the octamer and decamer which visually appears to preserve the overlap for their respective dimeric partners. Indeed, a structural alignment of the A-type dimers between the octamer and decamer gives a rmsd of 1.065 Å. Figure 4F shows the same monomers aligned, but in this case, their respective B-type interface with the dimeric partner from the opposite side shown. Here, one can see that the overlap is poor which is also reflected as a much larger rmsd of 3.137 Å for the alignment of octameric and decameric B-type dimeric partners. In order to more directly compare the interfaces, each monomer is simplified to the 2 Cα's of conserved leucine residues at its core, for example, Leu 102 and Leu139 from *Pv*Trx-Px1and corresponding Leu96 and Leu133 from *Py*Trx-Px1 (Figure 5A, B, respectively). An analysis of the changes between the *Py*Trx-Px1 octamer and the *Pv*Trx-Px1 decamer was undertaken using these conserved core residues as a representation of each monomer. Indeed, the model is validated, as the distance between the selected leucine residues (termed core length) is conserved throughout each of the structures and is similar between the two structures (Figure 5D). The orientation (termed angle between the dimers) and distance between the dimers within the oligomer is also conserved between the *Py*Trx-Px1 octamer and *Pv*Trx-Px1 decamer (Figure 5D). On the other hand, the orientation (termed angle between monomers) and distance between the monomers of the dimer (termed core distance) is dramatically different between the octamer and decamer. The observation that the different oligomerization is attributable to the interface within the dimer suggests that the oligomerization is not an artifact of crystallization and that the dimer itself is unique at least in terms of its B-type interface.

**Figure 6:**
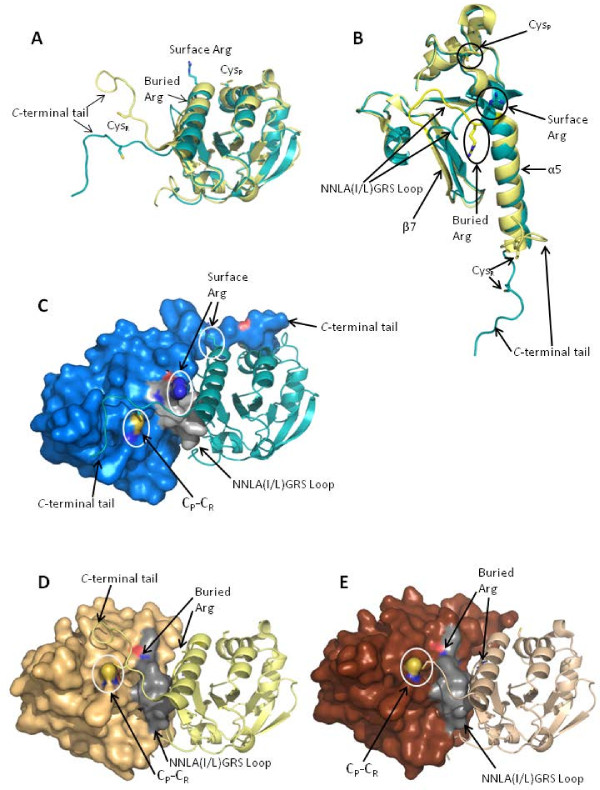
**Role of *C*-terminal tail and NNLA(I/L)GRS-loop in the determination the oligomerization of *Py*Prx-Tr1**. **(A) **and **(B) **Two views of the oxidized monomers of *Py*Trx-Px1 (blue) and *Pv*Trx-Px1 (yellow) showing their structural differences: *C*-terminal tail adopts a different orientation in each structure, NNLA(I/L)GRS-loop also adopts a different orientation in each structure (see panel B), and the Arg adopts a surface position in *Py*Trx-Px1 and a buried position in *Pv*Trx-Px1. Note that chain I from PDB: 2H66 is used because it is most complete at the *C*-terminus. As well, portions of the structures in **(B) **are hidden to facilitate the view of the loop (including up to Tyr42 from the *N*-terminus and from Ala56 to Asp 77). **(C) **In order to demonstrate how the *C-*terminal tail positions itself in the dimer, a hybrid surface-cartoon rendering of *Py*Trx-Px1 shows the orientation of the *C*-terminal tail and the NNLA(I/L)GRS loop in grey. **(D) **and **(E) **For comparison, the hybrid surface-cartoon renderings of *Pv*Trx-Px1 show the orientation of the *C*-terminal tail and the NNLA(I/L)GRS loop in grey. Note that **(D) **is from chains I and J which only shows a complete *C*-terminal chain for I. As well, for **(E) **chains C and D are used which both truncate around C_R _for the *C*-terminal tail, but show that the *C*-terminus is the same up to this point.

Analysis of the structures of *Py*Trx-Px1 and *Pv*Trx-Px1 identifies several key points of difference between the structures that may account for difference in oligomerization. First of all, the presence of a hydrogen bond between the dimers of *Py*Trx-Px1 (and not *Pv*Trx-Prx1) was already discussed (Figure 4C, D). Secondly, the *C*-terminal tails have different orientations, such that they are binding at different positions on the surface of their respective dimeric partners (Figure 6). The tails are bound by an intermolecular disulfide between C_P _and C_R_, as well as a series of hydrophobic interactions. The different binding orientations are linked to the orientation of the side chain of a conserved arginine (Arg142 and Arg148 for *Py*Trx-Px1 and *Pv*Trx-Px1, respectively) (Figure 6A, B). In the case of *Pv*Trx-Px1, the arginine is buried and the *C*-terminal tail adopts the typical binding pattern on the surface of its respective dimeric partner (Figure 6D). As opposed to *Py*Trx-Px1 where the equivalent arginine side chain is at the surface of the protein obstructing the typical pathway, such that the *C*-terminal tail adopts a different position at the surface of its partner (Figure 6C). Using the NCBI Molecular Modeling Database http://www.ncbi.nlm.nih.gov/Structure/MMDB/mmdb.shtml, a search was performed to identify 3D structures to similar *Py*Trx-Px1. A manual inspection of each the 2-Cys peroxiredoxins of the 143 low redundancy hits (from the 1532 total hits) showed no similarity in orientation to the *Py*Trx-Px1 *C*-terminal tail, as expected as no other toroid octameric peroxiredoxins are known to date. A second point of differentiation between the two structures is a loop with the conserved sequence ^142^NNLA(I/L)GRS^149 ^(numbering from *Pv*Trx-Px1 (PDB: 2H66)) that connects α5 and β7 (β-sheet involved in the peroxiredoxin B-type interface) and contains the afore mentioned buried/surface Arg. The loops from each structure adopt different conformations that put Arg142 at the surface for *Py*Trx-Px1 and the corresponding Arg148 buried for *Pv*Trx-Px1. Although the *Pv*Trx-Px1 structure is from a full-length construct, only a structure with an *N*-terminal truncation of 6 residues crystallized sufficiently well for data collection in the case *Py*Trx-Px1. Although both constructs bear a *N*-terminal His_6_-tag, the tag could only be cleaved from the *Pv*Trx-Px1 construct. Even after several days at room temperature (where typical conditions require an overnight reaction at 4°C), the His_6_-tag remained intact on *Py*Trx-Px1. Although the tag is partially visible in *Py*Trx_Px1 case(^-3^AFQG^1 ^from PDB: 2H01), it is not close enough to the conserved loop in both cases to affect its orientation. Further studies will determine what roles the structural features identified herein (*i.e*. the hydrogen bond between the dimers, the *N*-terminal tail, the surface/buried arginine, the NNLA(I/L)GRS-loop, and the *C*-terminal tail) play in the stoichiometry of oligomerization of these Trx-Px1 enzymes.

### Crystal structure of *Py*Prx6 with product bound

The *Py*Prx6-SO_2_H crystal structure has been solved at 2.3 Å (PDB ID: 3TB2). It is 47% identical to its closest human orthologue and shares 76% sequence identity with its *P. falciparum *orthologue. The closest available structure by sequence (at 48% sequence identity) is *Arenicola marina *peroxiredoxin 6 which was further identified as a 2-Cys Prx [34], but it also shares 47% sequence identity with a solved structure for a human 1-Cys Prx [35]. Overall, the human (PDB ID: 1PRX) and *P. yoelii *structures are very similar with a core thioredoxin-fold and a *C*-terminal domain connected by both an extended helix α5 and a loop. The core rmsd is 1.38A for 401 aligned residues in both structures. The *C*-terminal domain of *Py*Prx6 that comprised of 3 β-strands and a α-helix is larger than the *C*-terminal domain from the Prx1 subfamily (Figure 1D). This domain from each monomer extends over the other forming a domain swapped dimer.

**Figure 7:**
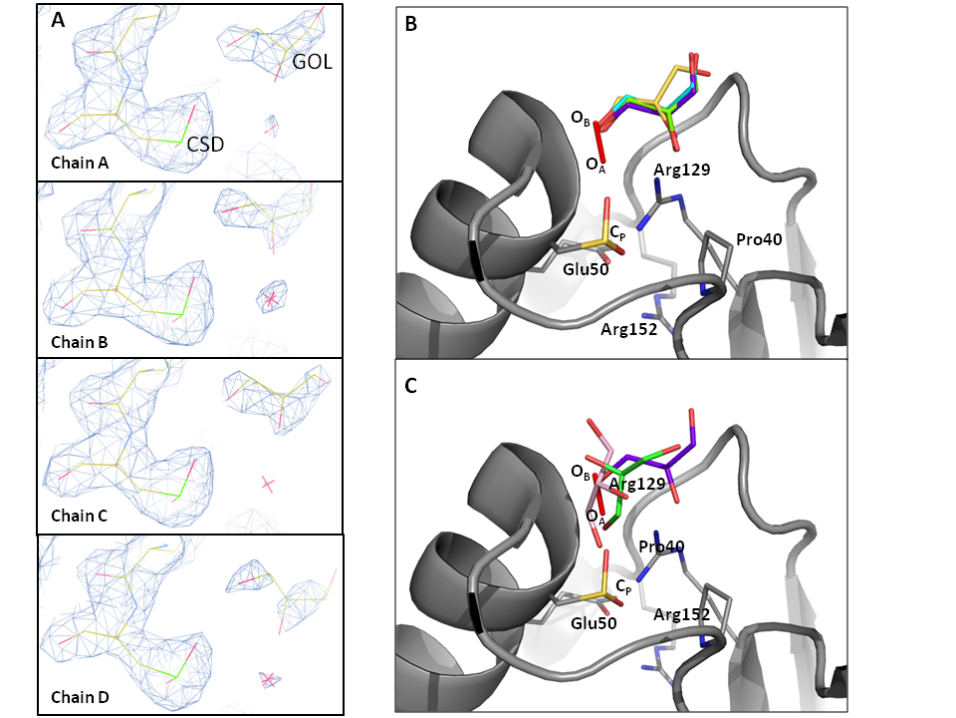
***Py*Prx6 active site depicting the sulfinic acid and the binding orientations of glycerol**. **(A) **Fc-Fo omit map (1.62 σ) of the active site from the 4 chains within the asymmetric unit of *Py*Prx6 (PDB: 3TB2) showing the density assigned to both the sulfinic acid of C_P _and the glycerol bound in the active site. Glycerol and sulfinic acid are labelled as GOL and CSD, respectively. Note that the carbons are yellow, sulfur is green, oxygen is red, and nitrogen is blue. **(B) **A comparison of the binding orientations of each of the glycerol molecules of *Py*Prx6 relative to H_2_O_2 _derived from an alignment with the *Ap*Trx-H_2_O_2 _structure (PDB: 3A2V) with our *Py*Prx6 structure. The H_2_O_2 _is shown as the red oxygen atoms with positions O_A _and O_B _labelled, while the 4 glycerol molecules in the *Py*Prx6 structure are shown with different coloured backbones and red oxygen atoms. Note that in each case, the terminal oxygen of the glycerol molecule aligns with position O_B_. **(C) **For comparison, from a similar structural alignment the known binding orientations for glycerol (from PDB: 3A2W (*Ap*Trx, chains A and C with the glycerol molecules depicted in pink and green, respectively) are compared to the same binding of H_2_O_2 _(PDB: 3A2V). Note that for *Ap*Trx chain A, the glycerol terminal oxygen aligns with position O_A_, while the glycerol molecule from *Ap*Trx chain C has oxygen atoms aligning with positions O_A _and O_B _as was previously reported [37] All structural alignments derived from the alignment of the following conserved residues PxxxxTxxC_P_, as was done previously [37].

The active site C_P _(Cys47) is located at the bottom of a narrow pocket (~4 Å by ~7 Å) at the end of helix α2. There are 2 additional densities in each of the 4 active site pockets of the asymmetric unit (Figure 7) which are best filled by a glycerol molecule (from the purification buffer used which contained 5% glycerol) and fitting the C_P _residues to their sulfinic acid derivatives (C_P_-SO_2_H). Previously, the C_P _has been structurally characterized in the whole range of oxidation states (including C_P_-SH, C_P_-SOH, S_P_-SO_2_H, C_P_-SO_3_H, and C_P_-SS-C_R_) [36]; and our data agrees with those sulfinic acid structures previously studied. As well, nearly 20 peroxiredoxin structures have either substrate or what has been termed substrate analogue bound in their active site pockets, including H_2_O_2_, benzoate, acetate, dithiothreitol (oxidized), ethylene glycol, glycerol, sulfate, citrate, and formate [36].

In order to understand the reaction mechanism, our focus is on the comparison of structures with substrate (H_2_O_2_) or glycerol bound. The binding of H_2_O_2 _to *Aeropyrum pernix *Tpx (thiol peroxidase) has been structural characterized (PDB: 3A2W and 3A2V) [38]. Further work on these structures and comparisons to other ligand bound peroxiredoxin structures previously showed that oxygen atoms of the ligands overlap with the proximal (relative to C_P_) oxygen atom (O_A_) and/or the distal oxygen atom (O_B_) of bound H_2_O_2 _[37]. Interestingly, the glycerol molecule found in these structures can adopt all three possibilities: (1) in one monomer of *Ap*Trx (PDB: 3A2W) it is found with a single oxygen atom overlapping O_A _(Figure 7C, pink); (2) in another monomer of *Ap*Trx (PDB: 3A2W), it is found to overlap with both O_A _and O_B _(Figure 7C, pink); and (3) reported for the first time, in our structure of *Py*Prx6, the glycerol is observed to overlay with only O_B _(Figure 7C, purple). Previously, only 2 anionic ligands (sulfate and citrate) were observed to occupy O_B _alone (see PDB: 1TP9 and 3DRN) [37]. Despite variations in the backbone orientation of the glycerol in our structure (which is also seen in the *Ap*Trx glycerol bound chains and presumably due to its conformational flexibility), the binding of all 4 glycerol molecules shows that each binds with the terminal hydroxyl in a similar position overlapping with O_B _site (Figure 7B). Oxygen atoms at position O_A _are postulated to be a mimetic for the substrate bound in a Michaelis complex ready for attack by the nucleophilic C_P_, while oxygen atoms at position O_B _are indicative of the leaving group (H_2_O or alcohol) [37]. As such, it can be suggested that the glycerol positioned with its terminal hydroxyl at O_B _is in a product bound configuration. Although there is variability in the binding of the remainder of the glycerol molecule, the alkyl group is always directed away from the active site pocket and the oxygen of the leaving group is in close proximity to a conserved threonine (Thr44). This arrangement suggests that it may be the proton donor, although others have suggested that this threonine functions as a hydrogen bond acceptor as it deprotonates the incoming substrate for attack by the C_P _thiolate and that bulk solvent is responsible for the protonation of the leaving group [37]. Therefore, the active site pocket is adapted to accommodate different substrates (and thus products), which is exemplified through the structural flexibility exhibited in the product bound glycerol shown herein. As well, the O_B _position appears to be designated for the oxygen of the leaving group and is well positioned for protonation by a conserved threonine.

**Figure 8:**
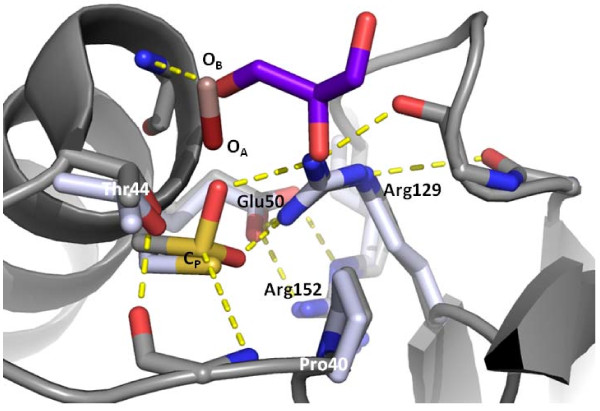
**Comparison of the active sites of *Py*Prx6•glycerol with *Ap*Trx•H_2_O_2_**. A view into the active site from above shows select main chain and side chain residues of *Py*Prx6 (PDB: 3TB2) in dark grey (with the associated glycerol shown in purple/red). In light grey the corresponding *Ap*Trx (PDB: 3A2W) side chain residues are shown (with the associated H_2_O_2 _shown in pink/dark red to differentiate O_A _and O_B_). The structural alignment is derived from the alignment of the following conserved residues PxxxxTxxC_P_, as was done previously [37].

The conserved residues of the C_P _loop comprised of ^40^PxxxxTxxC_P_^47 ^and conserved Arg127 (numbering refers to *Py*Prx6) are implicated in catalysis (Figure 8). For the conserved arginine in both *Py*Prx6 (Arg127) and ApTrx•H_2_O_2 _structures, it adopts position I, typical of Prx6, which bears a conserved arginine (Arg152) and glutamate (Glu50) (or possibly a glutamine or histadine) and supports the positioning by a hydrogen bonding network, as previously described [37]. The active site geometry is fully folded (FF) and is virtually identical to that of the *Ap*Trx•H_2_O_2_. As was recently described, the nucleophilic C_P _(as activated by a main chain amide N-H and the conserved arginine guanidinium) is expected to act as a thiolate and attack the substrate in an S_N_2 fashion [37]. These hydrogen bonds, as well as those secondary ones from the backbone carbonyls and the glutamic acid/second conserved arginine, surrounding the C_P _are preserved between the *Ap*Trx x•H_2_O_2 _and the *Py*Prx6•glycerol structures suggesting that the sulfinic acid form may be activated, and thus sufficiently nucleophilic (similar to its full reduced state) within the active site to undergo a further reaction with substrate. This would result in a subsequent oxidation of the active site to the sulfonic acid. Indeed, this form of the C_P _has been structural characterized in other cases (PDB: 2CV4, 2NVL, and 1XIY). The conserved proline serves as a barrier to solvent, while the main chain of the C_P _loop provides hydrogen bonds to the C_P _and the conserved threonine. Although at a reduced efficiency owing to the relative activity of an oxidized thiol relative to a thiolate, the preservation of the hydrogen bonding network about the active site cysteine, indicates that sequential H_2_O_2 _reductions are possible.

## Discussion

*Plasmodium *lacks the two major antioxidant enzymes of eukaryotic cells, namely glutathione peroxidase and catalase [39]. As such, the parasite is likely to rely on the peroxiredoxin family to control peroxide production (as well as other reactive oxygen and nitrogen species) during critical stages of its lifecycle, for example during erythrocyte invasion when heme metabolism and immune response pathways ensue. During the trophozoite stage (feeding period), Prx enzymes accounts for 0.5% of the total expressed protein [40]. One recent study suggests that *Pf*nPrx, the *P. falciparium *nuclear peroxiredoxin might be essential in the erthyrocyte stage, as neither *P. falciparum *or *P. berghei *knock-out lines could be generated despite several attempts and success with generating tagged nPrx-GFP fusion cell lines [14]. Analysis of the growth of a *P. berghei *Trx-Px1 knockout also suggests that Trx-Px1 is not essential for growth, but *P. falciparum *and *P. bergei *show differences in their lifecycles [41]. There is a possibility that Trx-Px1 is essential during the asexual growth of *P. falciparum *[31]. Expression of *Pf*1-Cys Prx is elevated during the trophozoite and early schizont stages (when the parasites are maturing during the liver phase) suggesting that this subfamily detoxifies ROS, like those released during heme metabolism [40]. Despite the important roles of peroxiredoxins, whether inhibitors targeting *Plasmodium *peroxiredoxins will lead to parasite death remains to be determined.

Structural characterization of the 2-Cys Prx enzymes has shown that the *C*-terminal tail (referred to as the C_R_) is essential for stabilizing the octameric/decameric arrangement of peroxiredoxins. When Trx-Px1 (or another 2-Cys Prx) is oxidized to the disulfide form, the C_R _loop is unfolded; and structural support of the octamer/decamer/dodecamer interfaces are weakened giving rise to dimer formation (as seen in part when our oxidized *Pf*Trx-Px1 is run on a gel filtration column). When the C_R _loop is folded as in the reduced form, the higher order oligomer is favoured because of increased stabilization for the B-type interface which supports oligomerization. The dramatic rearrangement of the *C*-terminal tail and ensuing changes in stability are clearly demonstrated in our structures from *Py*Trx-Px1 and *Pv*Trx-Px1 (both oxidized and reduced forms). However, the high concentrations of protein used in crystallizing the enzyme (and also apparently during most of our gel filtration experiments) may account for the trapping of the disulfide forms of these peroxiredoxins in their predicted unfavoured octameric/decameric/dodecameric forms. Factors associated with the oligomeric forms primarily include reduction of the active site disulfide, but also, high or low ionic strength, low pH, high magnesium or calcium concentrations, and overoxidation of the peroxidatic cysteine (Cys-SO_2_H) [28]. At physiologically relevant concentrations, peroxiredoxins can be expected to exist as dimers awaiting reduction; and upon reduction, the catalytic cycle is complete and the reduced peroxiredoxin oligomerizes [23]. With data from the novel octameric configuration of *Py*Trx-Prx1 studied herein, other specific structural features affecting oligomerization are considered. The molecular basis for octamer formation relies on the hydrogen bond between the dimers which is facilitated by the positioning of the *C*-terminal tail which in turn rests on interplay between the surface/buried arginine of the NNLA(I/L)GRS-loop and possibly the *N*-terminal tail. *In vivo *work directed at the further characterization of the different sizes and configurations of the oligomers will be necessary to fully understand the biological implications.

Aside from being antioxidant proteins, 2-Cys Prx (Prx1) has also been implicated in H_2_O_2_-mediated signal transduction. Eukaryotic 2-Cys Prx enzymes are sensitive to oxidative inactivation, while their bacterial orthologues are robust with respect to overoxidation [30]. The *Plasmodium *2-Cys Prx enzymes accordingly have the 3 sequence motifs indicative of the H_2_O_2_-sensitive peroxiredoxins, and as shown herein are structurally identical with respect to sensitivity to H_2_O_2 _to known H_2_O_2_-sensitive 2-Cys Prx enzymes, thus allowing low resting levels of H_2_O_2_, while permitting higher levels during signal transduction.

The *Py*Prx6 structure presented herein has greatly enhanced our understanding of the chemistry of the peroxiredoxins. With a product bound configuration, the residues supporting the leaving group are further understood. There is flexibility for the alkyl chain, but the O_B _position is indeed designated for the oxygen of the leaving group. As well, the retention of hydrogen bonds about the active site thiol (even in an oxidized state) indicates that it is poised for further reaction, albeit at a reduced efficiency owing to the reduced activity of an oxidized thiol relative to a thiolate.

## Conclusions

Our structural data and mass spectroscopy confirms that *Pv*Trx-Px1 is H_2_O_2_-sensitive peroxiredoxin. The characterization of the oligomerization of *Py*Trx-Px1 has identified structural features supporting its novel octameric oligomerization. Previously unreported abnormalities of the disulfide bond measurements in some of the Prx crystal structures are brought to the forefront. Finally, a crystal structure with an alcohol bound and the C_P _oxidized gives a view to the product bound complex providing insight into leaving group and the susceptibility of some peroxiredoxins to overoxidation. These results enhance our understanding of the structural variations of the peroxiredoxin oligomers and the nature of the catalysis by these remarkable enzymes. Further work will lend insight into the biological implications of the oligomerization and how to exploit the active site features in drug discovery programs.

## Methods

### Cloning, expression, and purification

Full-length *P. falciparum *Trx-Px1 encoded by PlasmoDB ID: PF14_0368 http://plasmodb.org/plasmo/[42] was cloned from *P. falciparum *3D7 genomic DNA with a His_6_-tag with an integrated thrombin cleavage site (MGSSHHHHHHSSGLVPR*GS). Full-length *P. knowlesi *Trx-Px1 encoded by PlasmoDB ID: PKH_126740 was cloned from *P. knowlesi *H genomic DNA with a His_6_-tag with an integrated TEV cleavage site (MGSSHHHHHHSSGRENLYFQ*G). Full-length *P. vivax *protein encoded by PlasmoDB ID:PVX_118545 with an *N*-terminal His_6_-tag and TEV cleavage site (as above) was cloned from a *P. vivax *Salvador I cDNA library (generously provided by Prof. Liwang Cui of Penn State University). Full-length *P. yoelii *protein encoded by PlasmoDB ID: PY00414 (Trx-Px1) was cloned from *P. yoelii *17XNL genomic DNA with an *N*-terminal His_6_-tag and integrated TEV protease site (as above). Full-length *P. yoelii *protein encoded by PlasmoDB ID: PY04285 (Prx6) was cloned from *P. yoelii *17XNL genomic DNA with an *N*-terminal His_6_tag with different integrated TEV protease site (MGSSHHHHHHSSGRENLYFQ*GHM) and *C*-terminal addition (GS). All enzymes were expressed and purified according to methods described previously [26] except for *Py*Prx6 which was expressed from Studier auto-induction media [27].

### Characterization

All mass spectra were completed on an Agilent LC-MS-TOF (Model #G1969A) running in positive ion mode and integrated with an Agilent 1100 series HPLC using an Agilent Poroshell 300-SB-C3 column for fast binding/elution desalting. All gel filtration experiments were complete on a AKTA purifier chromatography system (GE Healthcare Life Sciences) equipped with a Superdex S200 gel filtration column that was calibrated with 4 samples (bovine thyroglobulin (670 kDa), bovine γ-globulin (158 kDa), chicken ovalbumin (44 kDa), and horse myoglobin (17 kDa) all from Bio-Rad) in 10 mM HEPES, pH 7.4 and 500 mM NaCl.

### Crystallization and structure determination

*Pv*Trx-Px1_ox with the His_6_-tag intact was crystallized by mixing 1.5 μL of protein (at a concentration of 8 mg/mL in a buffer of 10 mM HEPES, pH 7.5, 500 mM NaCl) with 1.5 μL of reservoir solution containing 5% Peg 4 K, 50 mM NaAc, 100 mM NaAc, pH 4.6 in a hanging drop vapour diffusion setup with over 350 μL of reservoir solution at 18°C in VDXm plates (Hampton Research). Crystals appeared overnight and were flashed-cooled in liquid nitrogen (N_2(l)_) for data collection. Single wavelength data was collected at a synchrotron source (APS Beamline 17-ID) with a CCD detector (ADSC quantum 210). *Pv*Trx-Px1_red with the His_6_-tag intact was crystallized using the hanging drop vapour diffusion method in a VDXm plate with 350 μL of mother liquor at 18°C. 1.5 μL of the protein solution treated with 5 mM TCEP was mixed with 1.5 μL of the reservoir solution containing 19% PEG 3350, 150 mM lithium citrate. Crystals appeared overnight. Data for crystals flash frozen in N_2(l) _was collected at the synchrotron (APS Beamline 17-BM) with a CCD detector (MAR CCD 165 mm). *Py*Trx-Px1 with the His_6_-tag intact was crystallized at 4.3 mg/mL using the sitting drop vapour diffusion method in a Linbro plate with 300 μL of mother liquor at 18°C. 1.5 μL of the protein solution was mixed with 1.5 μL of the reservoir solution containing 1.6 M ammonium sulfate, 100 mM HEPES, pH 6.8, 200 mM NaAc, 20 mM NaBr, 5% ethylene glycol. Crystals appeared in 3-5 days and were flash frozen in N_2(l) _with data collected on a Rigaku FRE Superbright rotating anode with an RAXIS IV plate reader. *Py*Prx6 with the His_6_-tag intact at 15 mg/mL was crystallized by means of by hanging drop vapour diffusion in a VDXm plate. The plate was set with 1.5 μL protein plus1.5 μL buffer in each drop and 350 μL reservoir volume per well. Crystals emerged in 23% Peg 3350, 0.1 M Bis-Tris pH 5.5, 200 mM (NH_4_)_2_SO_4 _and 5% ethylene glycol at 20°C. MAD data from a crystal flash frozen in N_2(l) _was collected at the synchrotron (APS Beamline 17-ID) with a CCD detector (ADSC Quantum 4).

Data were processed using the HKL2000 package [43]. Each structure was solved by molecular replacement using modified homology models created with the FFAS03 program [44]. The structures were refined by iterative rounds of manual building in Coot [45] and refinement using refmac5 from CCP4 package [46]. All structures were refined with good statistics and geometry, checked with MOLPROBITY [47]. Final statistics and data information for each structure can be found in Table 3. Figures for structural models were created using the Pymol visualization software http://www.pymol.org.

**Table 3:** Data collection, phasing, and refinement statistics for the 2H66, 2I81, 2H01, and 3TB2

Structure	*Pv*Trx-Px1_ox	*Pv*Trx-Px1_red	*Py*Trx-Px1_ox	*Py*Prx6-SO_2_H
PDB Code	2H66	2I81	2H01	3TB2

Space Group	P21	C2221	P422	C2221

**Cell Dimensions**				

a (Å)	70.55	91.35	105.08	90.39

b (Å)	149.59	212.57	105.08	156.84

c (Å)	131.91	115.26	41.83	178.07

α (°)	90	90	90	90

β (°)	104.88	90	90	90

γ (°)	90	90	90	90

Wavelength	1.00	1.00	1.54178	0.97939

Resolution (Å)	50.00-2.48 (2.53-2.48)	48.28-2.45 (2.55-2.45)	50.00-2.30 (2.38-2.30)	20.00-2.30 (2.38-2.30)

Unique reflections	86441	41514	10337	53397

R_merge_	0.115 (0.488)	0.072 (0.460)	0.153 (0.471)	0.059 (0.397)

I/σI	20.18 (1.3)	15.51 (3.56)	26.55 (4.29)	24.04 (3.31)

Completeness (%)	99.2 (99.8)	99.9 (100.0)	94.6 (99.3)	99.7 (100.0)

Redundancy	3.4 (3.3)	6.4 (6.4)	13.9 (12.8)	4.3 (4.3)

**Refinement**				

Resolution (Å)	2.5	2.45	2.3	2.3

Number of Reflections	82137	39472	10337	53397

Test Set Reflection numbers	4302	2107	542	2858

R_work_/R_free_	0.194/0.232	0.217/0.265	0.208/0.232	0.186/0.207

Number of Atoms (protein/ligand/water)	13294/0/154	7190/0/80	1368/0/54	7055/89/364

Mean B_factor_	45.74	38.6	37.8	40.0

Ramachandran Favored (%)	95.37	95.21	91.86	98.01

Ramachandran Disallowed (%)	0.48	0.33	0.58	0.00

**RMS deviations**				

Bond length (Å)	0.0161	0.0083	0.0090	0.0086

Bond angle (°)	1.6765	1.2250	1.2343	1.1004

## Competing interests

The authors declare that they have no competing interests.

## Authors' contributions

WQ analyzed the structural data and solved the structures of *Pv*Trx-Px1_red and *Py*Trx-Px1, as well, identified and assigned previously unknown densities within the *Py*Prx6 structure. AD collected diffraction data on each of the structures and assisted in the solving of the structures. JCP helped analyze all of the structural data and provided insight into the oligomerization and the characterization of the active site of *Py*Prx6. AB solved *Py*Prx6. JM helped solve *Py*Trx-Px1. AKW solved *Pv*Trx-Px1_ox. TH investigated the *Pv*Trx-Px1 and *Py*Trx-Px1oligermization. RH conceived the study, provided insight, and helped draft the manuscript. JDA analyzed the structures, drafted the manuscript and figures, as well as purified, characterized, and crystallized both *Pv*Trx-Px1 structures and *Py*Trx-Px1. All authors read and approved the final manuscript.
